# New Type of Food Processing Material: The Crystal Structure and Functional Properties of Waxy and Non-Waxy Proso Millet Resistant Starches

**DOI:** 10.3390/molecules26144283

**Published:** 2021-07-15

**Authors:** Mengru Han, Ke Dang, Jiale Wang, Licheng Gao, Honglu Wang, Aliaksandr Ivanistau, Qinghua Yang, Baili Feng

**Affiliations:** 1 State Key Laboratory of Crop Stress Biology for Arid Areas, College of Agronomy, Northwest A & F University, Yangling, Xianyang 712000, China; hanmengru@nwafu.edu.cn (M.H.); dangke4718@163.com (K.D.); le80854634@126.com (J.W.); gaolicheng1995@163.com (L.G.); 2018050099@nwafu.edu.cn (H.W.); 2 College of Agronomy, Belarusian State Agricultural Academy, Michurin Street 5, 213407 Gorki, Belarus; ivanistov09@mail.ru

**Keywords:** proso millet, resistant starch, crystal structure, physicochemical properties, heat-moisture treatment, food applications

## Abstract

Resistant starch (RS) is widely used in the food industry because of its ability to regulate and protect the small intestine, but their distinct effects on the structural and functional properties of waxy and non-waxy proso millet starches are not completely understood. The crystalline structure and physicochemical properties of waxy and non-waxy proso millets’ starch samples were analyzed after heat-moisture treatment (HMT). The analysis revealed significant differences between the RS of waxy and non-waxy proso millets. The crystal type of proso millets’ starch changed from type A to type B + V. The relative crystallinity of the RS of waxy proso millet was better than that of non-waxy proso millet. The gelatinization temperature and thermal stability of RS significantly increased, and the pasting temperature (PTM) of the RS of waxy proso millet was the highest. The water solubility and swelling power of the RS in proso millet decreased, and the viscoelasticity improved. The correlation between the short-range ordered structure of RS and Δ*H*, and gelatinization properties has a stronger correlation. This study provides practical information for improving the nutritional benefits of waxy and non-waxy proso millet in food applications.

## 1. Introduction

Proso millet (*Panicum miliaceum* L.), commonly known as yellow rice, is one of the oldest crops in the world. It was domesticated in China for the first time about 10,000 years ago [1] and is currently an important food crop of northwest China [2]. It is still widely cultivated due to high resistance to stress, such as poor soil and drought. The proso millet grains contain several nutrients, including starch, protein, dietary fiber, vitamins, and mineral elements, and starch (58.5–73.5%) is the main carbohydrate [3]. Due to the nutritional value, proso millet is favored by people as a diet food, functional food, etc. The physicochemical properties of proso millet largely determine the eating quality [4], processing characteristics, and cooking process.

Natural starch (NS), a renewable and abundant polysaccharide, and is widely used in food production as a stabilizer, water-retaining agent. and thickener [4]. However, NS application is limited due to its imperfect and complex structure in functional and high-temperature food processing fields [5]. Therefore, NS is generally modified to eliminate the shortcomings and enhance the properties. Resistant starch (RS) is found in some natural foods (potatoes, rice, bananas) [6,7,8], also known as indigestible starch. Compared with NS, RS has properties similar to soluble fiber. Fan reported that the X-ray diffraction type of RS are mostly V-type or B-type with more stable structure, which increases the tolerance to α-amylase [5,9]. In addition, the specific surface area of resistant starch is small, so the chance of resistant starch contacting with enzymes is relatively small and the resistant starch cannot be easily digested by the enzymes in the small intestine of human [9]. However, as a new source of dietary fiber, RS has better properties than dietary fiber [10]. Studies have shown that the addition of RS delays the oxidation of lipids in foods and improves the emulsification ability [8,11]. RS has good crystal structure, viscosity stability, rheological properties and low water-holding power, which can meet the needs of the beverage industry [6]. RS can be used as a thickening agent in dairy products and soup [12]. Studies have reported that the intake of RS decreases postprandial glycemic and insulinemic responses, increases satiety [13], and improves physical fitness. These properties indicated the potential of RS for developing functional food. Nanoparticles of resistant starch can develop a new type of functional beverage rich in dietary fiber [7]. RS has higher gelatinization temperature and film-forming qualities than NS and improves the coating crispness of food [14]. RS can be added to bread as a texture modifier, so that the crust has good brittleness and softness [15]. Besides, we use heat–moisture treatment (HMT) to prepare food-grade RS, which is eco-friendly (no chemical residue) and suitable for food production [16,17].

Recently, the physicochemical properties and other aspects of starch of waxy proso millet were reported in detail by Chao et al. [18]. Besides, the genetic diversity and the inter-relationship of starch traits of proso millet varieties were analyzed based on genotype data [19]. Several studies have reported the RS content of crops such as wheat and rice [20]; however, few studies exist on RS of proso millet. The food market demands diversification for today’s people’s needs, and proso millet as a new food resource is less developed and has tremendous potential for development as a functional food. After HMT, analysis of the RS of proso millet will help understand the nutritional characteristics of proso millet as a functional food. Therefore, the study analyzed starch samples obtained from the grains of waxy and non-waxy proso millets, using beans (pea) and tubers (potato) as the controls. The study evaluated the structural, rheological, thermal, and gelatinization properties of HMT starch particles from waxy and non-waxy proso millet. The findings provide a foundation for using proso millet’s RS in the food industry.

## 2. Materials and Methods

### 2.1. Materials

Waxy proso millet (Shanmi1), non-waxy proso millet (Chimi2), and pea (Xiwan1) were harvested in 2020 at the Northwest A&F University’s experimental site (37°56′26″ N, 109°21′46″ E), Yu’lin Institute of Agricultural Sciences, Shaanxi Province, China. In the experiment, management was conducted according to local production practices and crop requirements. The potato was bought from the supermarket. Pea and potato served as controls in this study.

### 2.2. Starch Isolation and Purification

Starch was extracted from proso millet following the alkaline soaking method [4]. The seeds of waxy and non-waxy proso millet were fully hulled and ground into powder using a milling machine. The powder was dissolved in 0.2% (*w/v*) NaOH suspension and incubated at room temperature for 12 h. The suspension was first filtered through a 100-mesh sieve and then through a 200-mesh sieve and centrifuged thrice at 4000× *g* for 10 min. The top yellowish layer was discarded, and the precipitate was dissolved in distilled water. Then, the suspension was neutralized with HCl (1 mol/L). After neutralization, precipitates were washed thrice with distilled water. Finally, the starch was dried at 40 °C for 24 h, and the dried starch was ground into powder and passed through a 100-mesh sieve. The starch was put into self-sealing plastic bags and numbered for later use. The starches from pea and potato were separated according to the method of Joshi [21] and Wu et al. [22].

### 2.3. Preparation of RS

The four RS (R-Shanmi1, R-Chimi2, R-Pea, R-Potato) was prepared from proso millet, pea, and potato following the method by Gao et al. [23]. A 10% (*w/v*) emulsion of NS was prepared from four samples. The sample of 10% (*w/v*) emulsion was heated in an high-pressure sterilization at 121 °C for 30 min and placed at 4 °C overnight; this step was repeated twice. Then, the obtained sample was dried at 60 °C and sieved through an 80-mesh sieve to obtain the RS.

### 2.4. Analysis of Starch Granule

The morphology of NS and RS granules was observed using a scanning electron microscope (SEM; Nano SEM-450, American FEI Company, Hillsboro, OR, USA) at an acceleration potential of 5 kV [24]. Dried starch samples were pasted on the loading surface platform of SEM with double-sided tape and sputtered with gold. The microscopic magnification is 3000× for NS and 800× for RS. The particle size of NS and RS was measured using a laser diffraction instrument (Mastersizer 2000, Malvern, England). A small amount of starch was suspended in distilled water. The parameters were set according to the method of Cai et al. [25].

### 2.5. Color Characteristics

Color characteristics of NS and RS were measured using a colorimeter (Colorimeter Ci7600, X-Rite, Inc., Grand Rapids, MI, USA). L* value represents the lightness (+L: black, −L: white), a* (+a: red, −a: green), and b* (+b: yellow, −b: blue). The parameters of L*, a*, and b* were measured following the method by Wang et al. [26]. The total color difference (ΔE*) was calculated as follows:

$$\Delta E^{*}=\sqrt{L^{*2}+a^{*2}+b^{*2}}$$

### 2.6. Analysis of Starch Amylose Content 

The amylose content of the NS and RS was determined by the iodine-binding method by Kim et al. [20]. About 10 mg of starch was added into a 15 mL centrifuge tube with 1 mL of 1 mol/L NaOH and 100 µL of anhydrous ethanol. The mixture was placed in a water bath at 35 °C for 30 min for complete gelatinization, and then 8.9 mL of distilled water was added. Then, 200 µL of the concentrate taken in a 15 mL centrifuge tube was neutralized with 200 µL of HCl (0.1 mol/L). Then, 200 µL of iodine reagent and 9.4 mL of distilled water were added, shaken well, and placed at room temperature for 20 min. The amylose content of the mixture was determined by recording the absorbance at 620 nm using a visible light spectrophotometer [23].

### 2.7. X-ray Diffraction (XRD) 

The crystalline structure of starch was analyzed using XRD (D/Max 2550 VB +/PC, Rigaku Corporation, Rigaku, Japan) according to the method described by Liu et al. [27]. The diffractometer was operated at 40 kV and 100 mA, with the scattering angle range set at 5°–50° (10°/min) [28].

### 2.8. ATR-FTIR Spectrum Analysis 

The short-range ordered structure of NS and RS samples was analyzed by ATR-FTIR (Attenuated total reflection-Fourier transform infrared) spectroscopy (7000, Varian, Palo Alto, CA, USA). The values of 1045/1022 cm^−1^ and 1022/995 cm^−1^ were calculated from the obtained original spectra.

### 2.9. Water Solubility and Swelling Power of Starch

Water solubility and swelling power of starch were determined through the method of Wang et al. [26]. The 0.03 g of starch was mixed with 10 mL of distilled water in a 15 mL centrifuge tube, it was placed in a shaking water bath at 75, 85, and 95 °C for 30 min to prepare starch gel. The gelatinized sample was cooled to room temperature and centrifuged at 3000 r/min for 20 min, and the obtained supernatant was poured into an aluminum can and dried at 105 °C until the quality remained unchanged. Finally, the dry supernatant and precipitate were weighed. The water solubility and swelling power was calculated as follows:

$$\mathrm{Water}\;\mathrm{solubility}\;\left(\%\right)=\frac{A}{W}\times 100$$

$$\mathrm{Swelling}\;\mathrm{power}\;\left(g/g\right)=\frac{P}{\left(W-A\right)}\times 100$$

where A is mass of dried supernatant, P is mass of precipitated starch, and W is mass of dry starch.

### 2.10. Thermal Properties

The differential scanning calorimeter (TA Q2000, Perkin Elmer Instruments, New Castle, DE, USA) was used to determine the starch thermal properties. Approximately 3 g of NS and RS was mixed with 9 µL of distilled water in an aluminum DSC pan, and the mixture was allowed to sit for 12 h at 4 °C [29]. An empty aluminum DSC pan for reference. The mixture was then heated from 30 to 100 °C at the rate of 10 °C/min [24]. The gelatinization onset (*T*_o_), peak (*T*_p_), and completion (*T*_c_) temperatures and the gelatinization enthalpy (Δ*H*) of NS and RS was calculated using the universal Analysis 2000.

### 2.11. Pasting Properties of Starch

Approximately 3 g of the sample from NS and RS with 14% water content were weighed and mixed evenly with 25 mL of distilled water. A rapid viscosity analyzer (RVA4500, Perten, Stockholm, Sweden) was used to determine the gelatinization and pasting characteristics of the NS and RS. The peak viscosity (PV), breakdown viscosity (BD), final viscosity (FV), setback viscosity (SB), and gelatinization temperature (PTM) and peak time (PT) of starch were analyzed using the program parameters set according by Li et al. [19].

### 2.12. Rheological Properties of Starch

The DHR-1 rheometer (Waters Corporation, Milford, MA, USA) was used to determine the dynamic rheological properties of NS and RS. Here, the parallel metal plate of rheometer diameter was set at 40 mm and the gap at 1000 μm. About 0.4 g of starch was mixed with 5 mL of distilled water in a 10 mL centrifuge tube and gelatinized in a boiling water bath for 10 min. The mixture was loaded onto the rheometer plate, and the edges of the gap were covered with a layer of dimethylpolysiloxane (50 cP viscosity) to reduce evaporation. The experimental conditions were modified as described by Kong et al. [30].

### 2.13. Statistical Analysis

All measurements were performed in triplicate and data are represented as the mean ± standard deviation. All data were analyzed using variance analysis (SPSS17.0, Inc., Chicago, IL, USA). Differences between samples were considered statistically significant at *p* < 0.05.

## 3. Results and Discussion

### 3.1. Analysis of Starch Granules and Color

#### 3.1.1. Scanning Electron Microscopy (SEM) and Size Distribution of Starch Granules

**Figure 1:**
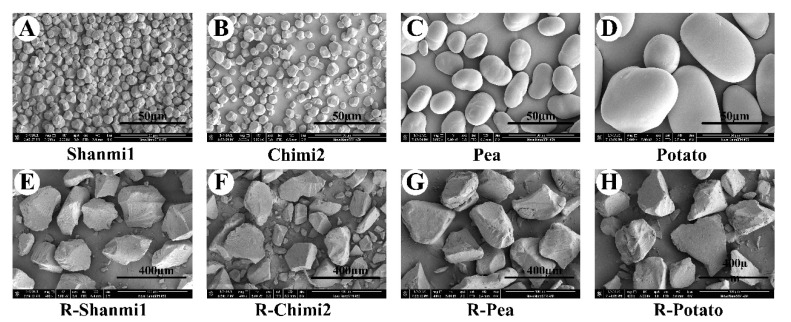
The morphology of NS (natural starch) and RS (resistant starch) granules of proso millet, pea, and potato were observed by SEM (note: the microscopic magnification is 3000× for NS, the microscopic magnification is 800× for RS). (**A**–**D**): NS of waxy proso millet, non-waxy proso millet, pea, potato, respectively. (**E**–**H**): RS of waxy proso millet, non-waxy proso millet, pea, potato, respectively.

Granule size and the morphology of NS and RS of proso millet, pea, and potato were observed by SEM (Figure 1). No significant difference was observed between waxy millet and non-waxy millet in starch granule morphology. Starch granules of proso millet were significantly smaller than those of pea and potato. The NS granules of proso millet were irregular polygons with prominent corners. Meanwhile, the pea and potato starch granules were mostly oval, while a few were spherical, with a smooth surface. Our observations on starch granules are consistent with the previous reports [4,31]; however, differences were observed between this study and the previous ones in granule size, which may be due to differences in crop variety [32], growing environment, and extraction method [4]. Furthermore, the granules of the four RS were similar in shape, and all the particles in RS were larger than those in NS. Meanwhile, the particle shape of RS was different from that of NS. Particles of RS were larger and uneven, with irregular gravel shapes, resulting in a more compact and stable structure, which improves its resistance to enzymatic digestion [13]. The huge difference between NS and RS was in the appearance and size of starch granules, this change may be due to the high-temperature gelatinization of NS, the expansion and degradation of the original starch particles to form a sticky paste, and the destruction of the NS structure [33]. 

**Figure 2:**
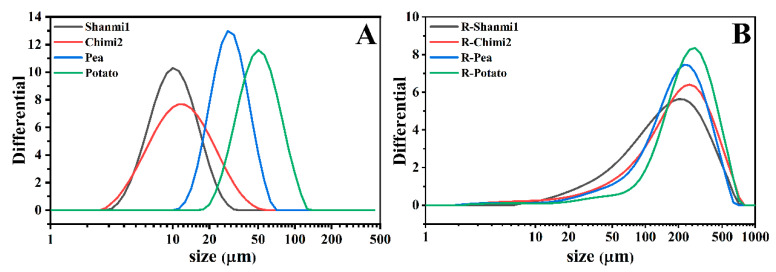
(**A**) Grain size distribution of NS (natural starch); (**B**) Grain size distribution of RS (resistant starch).

The particle size of starch affects the physicochemical properties, such as swelling power and rheological properties, as well as the functional properties of food [34]. The particle size distribution of NS and RS of proso millet, pea, and potato showed a single peak curve change (Figure 2). RS of potato had the largest granules among the eight starches, while NS of waxy proso millet had the smallest granules (Table 1). The NS of non-waxy proso millet had a slightly larger particle size than waxy proso millet and significantly smaller than pea and potato. The results of SEM of starches were consistent with these observations. However, the differences in the size between NS granules may be due to differences in genotype and growth conditions [35]. After HMT, the size of starch granules increased, and the particles of the RS appeared more concentrated. The RS granules of non-waxy proso millet were slightly larger than those of waxy proso millet after treatment. The difference between the particle size of RS of the waxy and non-waxy proso millet, pea, and potato became smaller. The diameter of RS was larger than NS, probably due to the destruction of the internal starch structure. Moreover, aging leads to the rearrangement of starch molecules, resulting in a compact structure with more significant crystallinity and lumpy particles [17]. The resistant starch of proso millet is suitable as a staple food processing material for people who lose weight. The RS is slowly digested in the human body and could improve the nutritional value of the staple food due to its larger granules [10].

#### 3.1.2. Color Measurement

Results of color analysis of the eight samples are shown in Table 1. Significant differences were observed in L* and total color between NS and RS (*p* < 0.05). The L* values of starches from the four NS samples ranged from 92.75 to 95.42 and from the four RS samples ranged from 81.86 to 89.11. The RS was light yellow compared with NS; the b* value of RS increased significantly, while the ΔE* reduced considerably upon treatment, probably because HMT induced chemical and physical changes in the thermally unstable substances of food, destroying the food color [36].

### 3.2. Amylose Content

The level of amylose content will have a certain impact on cooking quality and eating quality. The amylose content of NS and RS of proso millet, pea, and potato is shown in Table 2. The amylose content of NS ranged from 4.04 (waxy proso millet) to 37.85% (pea), and significant differences were observed among the different varieties. The NS amylose content of non-waxy proso millet (Chimi2) was 31.44%, which is higher than that of potato, but lower than that of pea. Previously, Yang reported an amylose content of 2.80 and 32.80% in the two types of waxy and non-waxy proso millet, respectively [4].

In general, the amylose content of RS was significantly higher than that of NS, indicating that RS has high temperature resistance and strong stretchability. Waxy proso millet (Shanmi1) RS had the lowest amylose content; the RS of non-waxy proso millet (Chimi2) was significantly higher than potato but lower than the pea. The amylose content of starch has an impact on food digestion. According to Riley’s research, starch with low amylose content gets easily digested [37]. Our findings indicate that RS of non-waxy proso millet (Chimi2) is more suitable for the human body with diabetes and obesity than that of waxy proso millet (Shanmi1).

### 3.3. Crystalline Structure of Starch

**Figure 3:**
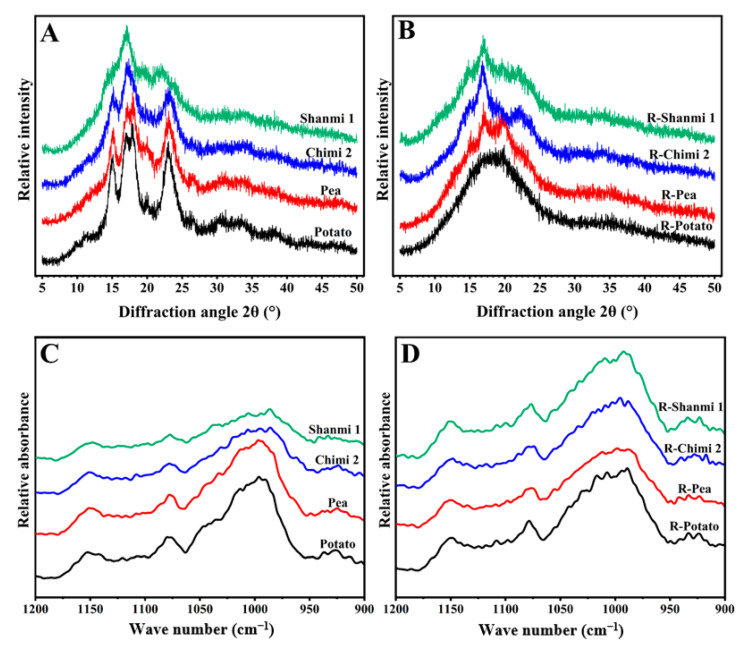
(**A**) X-ray diffraction patterns of natural starch (NS); (**B**) X-ray diffraction patterns of resistant starch (RS); (**C**) Ordered structure (FTIR) of natural starch (NS); (**D**) Ordered structure (FTIR) of resistant starch (RS).

XRD analyzed the structure characteristics of starch granules. Three crystalline types, A, B, and C, were identified in the plant starch-based on XRD peaks [38]. The XRD curves of NS and RS are shown in Figure 3A,B. Waxy proso millet and non-waxy proso millet showed A-type crystals, with a double peak at 17° and 18° 2θ, and two single peaks at 15° and 23° 2θ, consistent with the previous report [4]. Pea starch showed a typical C-type crystal [39], and the potato a B-type [34]. After HMT, the C-type crystalline structure of pea starch transformed into B type, and the A-type structure of proso millet starch transformed into B + V type crystal structure. The B + V type has a more stable double helix structure than other crystal types. Probably because after HMT, the amylose chain quickly changed to B-type crystal by low-temperature deposition, and at the same time, amylose reacts with lipids to form complexes and presents V-type starch crystallization, resulting in a stable crystalline structure [39]. Meanwhile, NS had no V-type crystalline structure, suggesting the absence of these complexes, formed only on heating [40]. Alternatively, these complexes may exist but are partially helical or short-range, producing the necessary XRD patterns [24].

The relative crystallinity of NS of waxy proso millet (Shanmi1), non-waxy proso millet (Chimi2), pea, and potato was 30.42, 28.64, 28.34, and 29.96%, respectively. The differences in relative crystallinity between different varieties of NS may be related to crop variety and amylose and amylopectin content [4]. After HMT, the relative crystallinity of RS increased (Table 2) due to the rearrangement of the double-helical crystallites of starch, resulting in an ordered crystalline matrix than that of NS [41]. These findings confirm that the crystal structure of proso millet RS is more stable than that of pea and potato, and that proso millet RS can better retain the nutritional value as a food processing raw material.

### 3.4. ATR-FTIR Analysis of Starch

At 1022 cm^−1^ (Figure 3C,D), the bands of waxy proso millet (Shanmi1) and non-waxy proso millet (Chimi2) starch were more evident than those of pea and potato starch. Between NS and RS, the waxy proso millet (Shanmi1) curve did not change, while that of the RS of non-waxy proso millet (Chimi2), pea, and potato changed significantly. The ordered starch structure was determined by the ratio of 1045/1022 cm^−1^ and the disordered structure by the ratio of 1022/995 cm^−1^ [40]. The 1045/1022 cm^−1^ ratio of RS decreased, while the 1022/995 cm^−1^ ratio increased compared with NS, indicating the gradual destruction of the ordered structure of waxy proso millet, non-waxy proso millet, pea, and potato starch during high temperature and pressure treatment (Table 2). The ordered structure significantly affected swelling power and pasting viscosity [42].

### 3.5. Dissolution Characteristics of Starch

**Figure 4:**
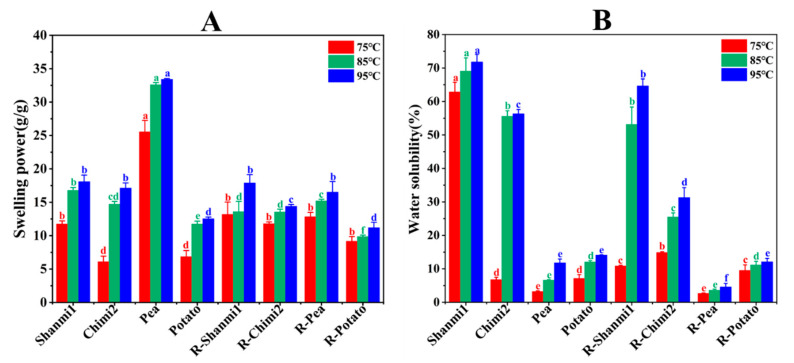
(**A**) Swelling power of NS (natural starch) and RS (resistant starch); (**B**) Water solubility of NS (natural starch) and RS (resistant starch). The error bars (a–f) indicate the standard errors of the means (*n* = 3).

The solubility and swelling degree of starch reflect the size of the binding capacity of starch molecules and water, which is determined by the water absorption capacity of starch granules and the binding degree of starch molecules and water molecules [18]. The water solubility and swelling power of NS and RS showed a similar trend at different temperatures (Figure 4). With the increase in temperature, the solubility and swelling power of NS and RS significantly increased. The water solubility and swelling power of waxy proso millet (Shanmi1) starch were higher than that of non-waxy proso millet (Chimi2) starch, consistent with the previous reports [4]. At 95 °C, the water solubility was 64.63, 31.27, 4.60, and 12.09% for RS from waxy proso millet (Shanmi1), non-waxy proso millet (Chimi2), pea, and potato, respectively. The crystalline structure of waxy proso millet starch was relatively stable. However, pea starch had the maximum swelling power, which may be related to the particle size, morphology, the ratio of amylose and amylopectin, and the ratio of long and short chains in amylopectin [26,31]. Besides, the results revealed a negative correlation between swelling power and amylose content (Significance, −0.927 **). Amylose inhibits starch granule swelling [25]. Waduge has reported that the decrease in water solubility of RS was probably due to the enhanced interaction and bonding between amylose and amylopectin molecules [43], which prevented amylose from leaching from granules [44]. Similar results also had been found in rice [45] and corn [46]. On the other hand, the decrease in swelling power may be due to the rearrangement of amylopectin, which led to more ordered double-helical side-chain clusters, making the particle structure more rigid. This also limited the particle expansion [47], leading to an increase in crystallinity. Thus, the high water solubility and swelling power of RS of waxy proso millet make it suitable for developing puffed and brittle food modifiers, improving the structural properties of food, and increase the expansion coefficient of extruded cereal and snack foods. In such a case, the puffed food made with RS will become soft and will not be broken after being soaked in milk and other beverages, which is more popular with consumers [2,48]. In addition, the quality of bread with resistant starch of proso millet is significantly better than that of bread with traditional dietary fiber. This is because the water absorption rate of resistant starch as a bread additive is lower than that of dietary fiber as a bread additive [49].

### 3.6. Differential Scanning Calorimetry (DSC)

The *T*_o_, *T*_p_, *T*_c_, and Δ*H* of NS from waxy and non-waxy proso millet, pea, and potato ranged from 61.05 to 74.70 °C, 64.68 to 78.61 °C, 73.55 to 88.82 °C, and 10.60 to 17.59 J/g, respectively (Table 3). The *T*_p_ and Δ*H* of NS of waxy proso millet (Shanmi1) were the highest, with higher crystallinity. However, the Δ*H* values and the crystallinity of pea starch were low, and the amylose content increased compared with proso millet and potato starch. These observations indicate that the thermal properties of starch are mainly related to crop species, crystalline type, and amylose content [3].

The *T*_o_, *T*_p_, and *T*_c_ of RS were significantly higher, while the Δ*H* was lower than NS in proso millet, pea, and potato (Table 3). The *T*_p_ of non-waxy proso millet was lower than that of waxy proso millet, and the Δ*H* of waxy proso millet was the highest. Higher gelatinization temperature indicates that RS is challenging to gelatinize and is more stable when used as an additive. Besides, a higher gelatinization temperature means a perfect crystal structure in the starch [50]. Studies have indicated that the gelatinization temperature increases due to the interaction between natural lipids and amylose complexes [51]. Meanwhile, this makes the starch granule stable and resistant [52]. The decrease in Δ*H* observed upon HMT in the present study may be due to the destruction of the crystalline and amorphous regions of the double-helical structure of starch [27]. Therefore, higher temperatures are needed to start gelatinization during RS processing in food products. In the food processing industry, the addition of RS, especially to macaroni and noodles, can increase its cooking resistance, help maintain a rigid structure, and avoid sticking [35].

### 3.7. Pasting Properties

As shown in Table 4, the pasting properties of NS and RS in waxy proso millet (Shanmi1), non-waxy proso millet (Chimi2), pea, and potato were significantly different. The PTM and PT of non-waxy proso millet were higher, indicating a low gelatinization degree [23]. The FV and SB of waxy proso millet starch were higher than those of non-waxy proso millet. However, the PV and BD of NS of non-waxy proso millet were lower than that of waxy proso millet, probably due to the higher starch content in the non-waxy proso millet. The FV of NS of pea was the highest. Meanwhile, potato starch had higher gelatinization viscosity, probably due to larger starch particles and higher expansion degrees, so potato starch is more suitable for pudding and confectionery industries [53]. These results indicated that the non-waxy proso millet starch had the highest stability in a hot paste. In contrast, the waxy proso millet starch had good gelatinization, stability (in cold paste), and anti-aging ability. Thus, non-waxy proso millet (Chimi2) starch appears suitable for high-temperature pot food, while waxy proso millet (Shanmi1) starch is ideal for frozen food [4].

The PV, BD, FV, and SB of RS were significantly lower compared with NS; waxy proso millet had the lowest among the different samples. It is possible that the cross-linking between amylose chains got strengthened after HMT, making it challenging to leach amylose [54]. However, the PV of starch decreased after treatment, indicating a decrease in water solubility and swelling power of starch during gelatinization, and the PTM of starch increased, consistent with the change in DSC gel temperature. Perhaps, the cross-linkages between amorphous areas were rearranged, forming ordered crystalline regions and starch molecules that demanded higher temperature for pasting [55]. Our observations indicated that RS was not easy to gelatinize and had high-temperature resistance and better gelatinization ability and thermal stability compared with NS. Thus, the RS of waxy proso millet can be used as a thickener in soups and dairy products due to high gelatinization temperature and low water retention [10]. For example, the RS of proso millet can be used in the development of functional yogurt. The RS of proso millet can increase the viscosity and foam stability [56] of yogurt and reduces the intake of sugar by the human body.

### 3.8. Dynamic Rheological Properties

**Figure 5:**
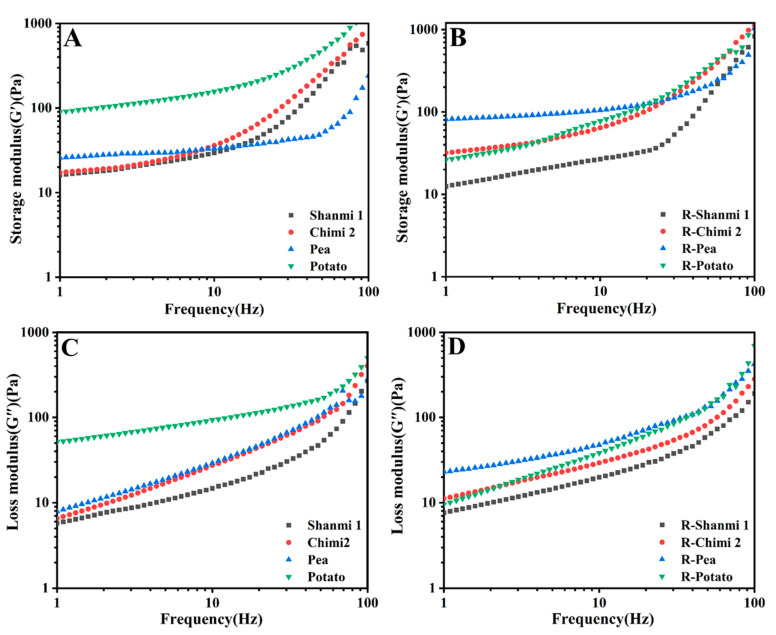
(**A**) Storage Moduli (*G’*) of natural starch (NS); (**B**) Storage Moduli (*G’*) of resistant starch (RS); (**C**) Loss Moduli (*G”*) of natural starch (NS); (**D**) Loss Moduli (*G”*) of resistant starch (RS).

Storage moduli (*G’*) and loss moduli (*G”*) were used to determine the elasticity and viscosity of starch. The *G’* and *G”* curves of the starches are shown in Figure 5. The *G’* of NS was greater than *G”*, and both *G’* and *G”* increased with the increase in frequency, indicating better elastic properties than the viscous properties and the dependency of *G’* and *G”* on frequency. When the frequency was greater than 16 Hz, the *G’* of RS of potato was the largest, and that of pea was the smallest, indicating greater potato elasticity and softer pea gel. The *G”* values of NS of potato and pea were higher than that of proso millet, and indicated that their viscosity was higher, being consistent with pasting properties results. The *G’* of RS of non-waxy proso millet (Chimi2) and pea was higher, indicating improved starch stability. The *G’* of RS in waxy proso millet (Shanmi1) was the lowest, indicating low elasticity due to low amylose content [57]. Besides, *G’* is directly related to the cross-linking density in the gel network. More rigid gels have limited application in food processing and may cause uneven mixing of processed materials as they require greater flow force [55]. The *G’* and *G”* values of RS in non-waxy proso millet (Chimi2) and pea increased compared with NS, probably because the crystalline structure of starch particles got destroyed by high pressure and high temperature. Moreover, the crystalline structure of RS was stable after HMT, and the flexibility of the starch gel improved [58]. The RS of proso millet is suitable for adding to the production of vermicelli to improve the taste and boiling resistance of vermicelli.

### 3.9. Correlation Analysis

**Figure 6:**
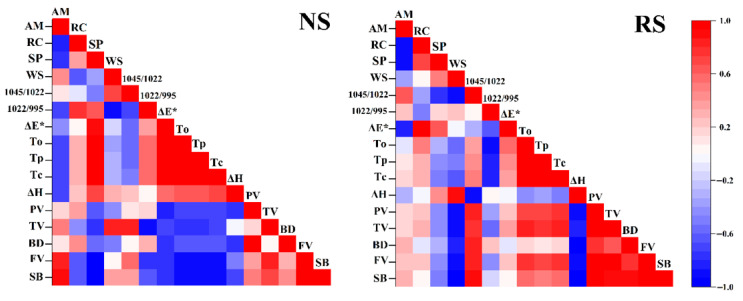
Pearson’s correlation coefficients between structural and physicochemical properties of the NS (natural starch) and RS (resistant starch) samples. WC, water solubility; SP, swelling power; RC, relative crystallinity; AM, amylose content.

Furthermore, a heat map was constructed to analyze the relationship between the amylose content of NS and RS and relative crystallinity, swelling power, water-solubility, thermal properties, and gelatinization properties. Amylose content positively correlated with FV and SB in NS and negatively correlated with relative crystallinity, swelling power, 1022/995 cm^−1^, *T*_o_, *T*_p_, *T*_c_, and Δ*H* (Figure 6). Meanwhile, the amylose content positively correlated with 1045/1022 cm^−1^ in RS and negatively correlated with relative crystallinity, swelling power, and ΔE*. The thermal performance of NS was negatively related to the gelatinization properties, while that of RS was closely related. However, the correlation between the short-range ordered structure of RS and Δ*H*, and gelatinization properties has a stronger correlation compared with NS. This indicates that the short-range ordered structure of RS promotes the gelatinization of starch, so that the functional properties of RS are not easily damaged in food processing.

## 4. Conclusions

This study analyzed the crystal structure and physicochemical properties of the RS of waxy and non-waxy proso millet. Differences in the effects of HMT on different properties between NS and RS were analyzed. HMT resulted in the rearrangement of starch particles and a larger size of grains. The A-type crystalline morphology and short-range ordered structure of proso millet starch changed after HMT. Further, the A-type structure of waxy and non-waxy proso millet starch transformed into B + V type, which was more stable than peas and potatoes. HMT increased the amylose content, relative crystallinity, gelatinization temperature, and gelatinization time of starch from the different plant sources. Meanwhile, water-solubility, swelling power, and gelatinization enthalpy reduced. The *G’* and *G”* values of RS in non-waxy proso millet increased. Therefore, RS has strong gelatinization ability and good thermal stability. These findings suggest that HMT rearranges the proso millet starch particles to form a more ordered and stable crystal structure than pea and potato. Therefore, in today’s pursuit of food diversification, the resistant starch of broomcorn millet can be used as a new type of food processing material, and has great potential for development in the food industry due to its strong structural stability. The study’s findings provide a theoretical basis for applying proso millet starch as an additive and in the production of new foods.

## Figures and Tables

**Table 1:** Granule size and color values of NS (natural starch) and RS (resistant starch) ^a^.

Varieties and Lines	Granule Size ^b^			Color Values ^c^			
	d (0.5) µm	D (3, 2) µm	D (4, 3) µm	L*	a*	b*	ΔE*
Shanmi1	9.91 ± 0.35 g	9.14 ± 0.31 g	10.73 ± 0.49 g	95.05 ± 0.14 b	0.20 ± 0.01 b	1.34 ± 0.08 d	95.06 ± 0.14 b
Chimi2	11.22 ± 0.23 g	9.60 ± 0.16 g	12.92 ± 0.09 g	95.42 ± 0.01 a	0.13 ± 0.01 c	1.59 ± 0.01 c	95.43 ± 0.01 a
Pea	26.95 ± 0.37 f	25.44 ± 0.32 f	28.39 ± 0.36 f	93.28 ± 0.16 c	0.29 ± 0.04 a	3.15 ± 0.02 a	93.33 ± 0.16 c
Potato	47.39 ± 0.14 e	44.36 ± 0.08 e	50.25 ± 0.36 e	92.75 ± 0.02 d	0.07 ± 0.01 d	2.37 ± 0.01 b	92.78 ± 0.02 d
R-Shanmi1	159.53 ± 3.87 d	86.52 ± 3.80 b	184.33 ± 8.67 d	89.11 ± 0.10 a	1.01 ± 0.01 b	5.65 ± 0.02 c	89.29 ± 0.10 a
R-Chimi2	183.32 ± 2.78 b	74.06 ± 0.91 d	205.38 ± 4.49 b	82.58 ± 0.18 c	2.54 ± 0.02 a	11.78 ± 0.03 a	83.46 ± 0.18 c
R-Pea	179.08 ± 3.36 c	80.75 ± 2.72 c	195.12 ± 2.93 c	81.86 ± 0.17 d	2.57 ± 0.08 a	11.56 ± 0.10 b	82.72 ± 0.15 d
R-Potato	232.50 ± 1.73 a	117.53 ± 4.95 a	262.65 ± 5.09 a	87.72 ± 0.13 b	0.36 ± 0.01 c	5.14 ± 0.03 d	87.87 ± 0.13 b

Note: ^a^ Data are means ± standard deviations. Values in the same column with different letters are significantly different (*p* < 0.05). ^b^ The d (0.5) is the granule size at which 50% of all the granules by volume are smaller. The D (3, 2) and D (4, 3) are the surface-weighted and volume-weighted mean diameter, respective. ^c^ L*, a*, b* represent the brightness, red-green, yellow-blue value of the sample color, respectively. ΔE* represents the comprehensive evaluation of the sample color.

**Table 2:** Amylose content, relative crystallinity, and IR ratio of NS (natural starch) and RS (resistant starch).

Varieties and Lines	Amylose Content (%)	Relative Crystallinity (%)	IR Ratio	
			1045/1022 cm^−1^	1022/995 cm^−1^
Shanmi 1	4.04 ± 0.19 h	30.42 ± 0.12 c	78.55 ± 0.88 b	75.72 ± 1.25 d
Chimi 2	31.44 ± 0.60 e	28.64 ± 0.08 g	73.02 ± 0.28 c	74.05 ± 0.86 e
Pea	37.85 ± 0.14 b	28.34 ± 0.08 h	83.21 ± 2.82 a	66.79 ± 0.44 f
Potato	27.88 ± 0.90 f	29.96 ± 0.08 e	77.82 ± 0.55 b	74.90 ± 0.66 de
R-Shanmi 1	7.55 ± 0.15 g	32.44 ± 0.06 a	66.08 ± 0.65 e	82.55 ± 0.98 b
R-Chimi 2	36.41 ± 0.40 c	30.14 ± 0.06 d	71.03 ± 0.76 cd	86.82 ± 0.92 a
R-Pea	46.77 ± 0.70 a	29.57 ± 0.09 f	69.52 ± 0.72 d	83.14 ± 0.46 b
R-Potato	33.08 ± 0.78 d	31.66 ± 0.09 b	72.75 ± 0.04 c	80.85 ± 0.92 c

Note: Data are means ± standard deviations. Values in the same column with different letters are significantly different (*p* < 0.05).

**Table 3:** Thermal properties of NS (natural starch) and RS (resistant starch).

Varieties	To (°C)	Tp (°C)	Tc (°C)	ΔH (J/g)
Shanmi 1	74.70 ± 0.14 d	78.61 ± 0.12 e	88.82 ± 1.50 e	17.59 ± 0.48 a
Chimi 2	72.77 ± 0.06 e	76.45 ± 0.38 f	86.24 ± 0.46 f	13.10 ± 0.15 c
Pea	61.05 ± 0.10 f	64.76 ± 0.07 g	74.62 ± 0.44 g	14.33 ± 0.16 b
Potato	61.10 ± 0.34 f	64.68 ± 0.35 g	73.55 ± 0.32 g	10.60 ± 0.14 d
R-Shanmi 1	132.24 ± 0.52 b	132.30 ± 0.54 c	133.79 ± 0.63 c	4.73 ± 0.01 e
R-Chimi 2	128.69 ± 0.84 c	129.13 ± 0.90 d	131.47 ± 0.66 d	2.45 ± 0.26 f
R-Pea	131.86 ± 0.07 b	133.78 ± 0.19 b	135.39 ± 0.37 b	4.38 ± 0.07 e
R-Potato	137.64 ± 0.09 a	138.35 ± 0.51 a	140.37 ± 0.22 a	1.41 ± 0.17 g

Note: Data are means ± standard deviations. Values in the same column with different letters are significantly different (*p* < 0.05). To, onset temperature; Tp, peak temperature; Tc, conclusion temperature; ΔH, gelatinization enthalpy.

**Table 4:** Pasting and thermal properties of NS (natural starch) and RS (resistant starch).

Varieties	PV (cP)	BD (cP)	FV (cP)	SB (cP)	PTM (°C)	PT (min)
Shanmi1	2574 ± 19.78 d	1285 ± 31.75 c	1633 ± 5.72 e	348 ± 6.24 f	77.04 ± 0.07 d	4.13 ± 0.00 g
Chimi2	1658 ± 13.53 e	630 ± 6.77 d	2984 ± 87.95 d	1957 ± 81.18 c	81.68 ± 0.03 a	4.30 ± 0.03 e
Pea	4464 ± 42.15 b	1719 ± 33.31 b	6208 ± 186.83 a	3392 ± 59.33 a	79.92 ± 0.03 b	4.67 ± 0.00 d
Potato	17562 ± 52.50 a	16582 ± 192.05 a	4652 ± 80.22 b	2944 ± 227.42 b	67.89 ± 0.05 c	2.87 ± 0.00 h
R-Shanmi1	415 ± 32.27 h	25 ± 5.20 e	547 ± 5.20 h	159 ± 32.27 g	83.78 ± 1.28 a	4.20 ± 0.00 f
R-Chimi2	1190 ± 8.85 f	536 ± 10.93 d	1406 ± 1.04 f	752 ± 1.04 e	80.81 ± 0.84 bc	6.75 ± 0.07 b
R-Pea	489 ± 8.85 g	47 ± 1.56 e	747 ± 6.24 g	305 ± 1.04 fg	83.19 ± 0.45 a	8.00 ± 0.00 a
R-Potato	2842 ± 7.29 c	525 ± 20.82 d	3528 ± 1.56 c	1217 ± 15.09 d	68.20 ± 0.44 e	5.03 ± 0.04 c

Note: Data are means ± standard deviations. Values in the same column with different letters are significantly different (*p* < 0.05). PV, peak viscosity; BD, breakdown viscosity; FV, final viscosity; SB, setback viscosity; PTM, Pasting temperature; PT, peak time.

## Data Availability

All relevant data are included in the article.

## Sample Availability

Samples of the compounds are available from the authors.
